# Endoscopic Treatment of Idiopathic Subglottic Stenosis: A Systematic Review

**DOI:** 10.3389/fsurg.2019.00075

**Published:** 2020-01-10

**Authors:** Emilie Lavrysen, Greet Hens, Pierre Delaere, Jeroen Meulemans

**Affiliations:** Otorhinolaryngology-Head and Neck Surgery, University Hospital Leuven, Leuven, Belgium

**Keywords:** balloon dilation, carbon dioxide laser, idiopathic subglottic stenosis, laryngotracheal stenosis, mitomycin C, Nd:YAG laser, rigid dilation

## Abstract

**Purpose:** To identify different endoscopic techniques for treatment of idiopathic subglottic stenosis (iSGS) and evaluate treatment results.

**Methods:** Embase and Cochrane Library were searched for publications on endoscopically treated iSGS. Identified interventions included procedures with cold knife, dilation (rigid or balloon), or laser (CO_2_ or Nd:YAG), used in several combinations and supplemented with mitomycin C and/or corticosteroids. Primary endpoint was time interval between successive endoscopic procedures. Secondary endpoints were stenosis recurrence rate, total number of interventions per patient during follow-up, tracheotomy rate, and rate of open surgery.

**Results:** Eighty-six abstracts were reviewed and 15 series were included in the analysis. Mean sample size was 57 subjects (range 10–384, σ 90.84) and mean age was 47 years (range 36–54, σ 4.45). Time interval ranged from 2 to 21 months [weighted mean (WM): 12]. Rate of stenosis recurrence ranged from 40 to 100% (WM: 68%). Mean amount of interventions per patient varied between 1.8 and 8.3 (WM: 3.7). Tracheotomy rate varied between 0 and 26% (WM: 7%) and rate of open surgery varied between 0 and 27% (WM: 10%). Single modality CO_2_ lasering showed highest rate of recurrence, highest amount of interventions, and shortest time interval. Combined techniques generated overall better outcomes.

**Conclusions:** A multitude of endoscopic techniques are being used for treating iSGS, all with a considerable recurrence rate. In this review, no superior modality could be identified. Consequently, endoscopic management could be considered a valuable primary treatment option for iSGS, but open surgery still plays an important role.

## Introduction

### Rationale

Subglottic stenosis is a condition that is characterized by the narrowing of the subglottic area and is most commonly caused by mechanical trauma after endotracheal intubation. Less frequently, stenosis of the subglottic area is the result of external trauma, heat trauma, external beam radiotherapy, surgery, upper respiratory tract infections, granulomatosis with polyangiitis (former Wegener's granulomatosis), amyloidosis, or collagen vascular diseases. A rare variant is the idiopathic subglottic stenosis (iSGS), defined as a subglottic stenosis of which the underlying cause cannot be determined (1). iSGS is a slowly progressive, non-specific inflammatory process that is mainly diagnosed in women aged 30–50 years and results in significant morbidity, such as respiratory distress and intolerance for physical activity (1, 2). The condition remains challenging for the treating physician, since both the pathogenesis and the treatment outcomes remain speculative. Both gastroesophageal reflux disease and autoimmune disease have been implicated in the etiology. More recently, hormonal factors are believed to play a major role, because of the invariable predominance in female patients. Nevertheless, various studies have failed to demonstrate estrogen receptors in these stenotic lesions (1, 3, 4). The optimal therapeutic management of the disease remains just as unsubstantiated (5). So far, many different strategies have been proposed, but no permanent curative solution has been uncovered. Endoscopic treatment techniques include radial incision or evaporization with a carbon dioxide laser (CO_2_ laser) or a neodymium-doped yttrium aluminum garnet laser (Nd:YAG laser), rigid dilation, balloon dilation, or cold knife scar excision. Generally, a combination of these endoscopic techniques is selected and supplemented with mitomycin C (MMC) and/or corticosteroids (CS) intralesional injections. Endoscopic techniques are an attractive initial treatment option for iSGS, since open techniques, such as cricotracheal resection, are more invasive and associated with a higher level of morbidity. The purpose of these management strategies is to establish airway patency without dyspnea complaints. However, many patients develop restenosis and require several interventions (6). Since the prevalence of iSGS is low and no treatment guidelines have been designed, comparative data on outcomes, and treatment success rates remain sparse (7). The uncovering of the ideal treatment strategy for this rare disease would guide clinical decision making and make the approach unambiguous.

### Objectives

This systematic review aims to identify and evaluate the existing literature concerning the endoscopic management of idiopathic subglottic stenosis.

### Research Question

Mapping of all the different endoscopic techniques and comparing their outcomes, is one technique superior to another?

## Methods

### Study Design

This study was reported as a systematic review based on the statements from the Preferred Reporting Items for Systematic Reviews and Meta-Analyses (PRISMA) guidelines (8). To define the objectives and methodology of this systematic review, an a priori protocol was established. The PICO question [population (P), intervention (I), comparison (C), and outcome (O)] was the following: “In adults with idiopathic subglottic stenosis, does any of the endoscopic management strategies have significantly superior results?”

### Systematic Review Protocol

The systematic review handled strict inclusion and exclusion criteria. The defined population was adults aged >18 years with the diagnosis of iSGS (8). Inclusion criteria comprised (1) etiology of stenosis as idiopathic; (2) endoscopic treatment; (3) paper written in English language; and (4) series including 10 or more patients. Exclusion criteria comprised (1) etiology of stenosis other than idiopathic or not listed; (2) site of the stenosis other than subglottic or not listed; (3) open surgical treatment of subglottic stenosis; (4) age < 18 years; and (5) case report or case series including <10 patients.

### Search Strategy

A systematic review of the literature was conducted using the databases of PubMed and Cochrane Library in March 2018. We searched for articles relating to patients with idiopathic subglottic stenosis (domain) and endoscopic treatment (intervention), using the following search words: “idiopathic subglottic stenosis endoscopic treatment.” Articles were excluded if the main subject was not in relation to the set domain in combination with the intervention or in case the language was other than English. References were screened for related articles and a general Internet search was conducted to verify that all significant articles were included. Subsequently all abstracts were screened for eligibility in the review. Articles written before the year 2000 and case reports were removed from the database, since the aim was to keep the review recent and the results as substantiated as possible.

### Data Sources, Studies Selection, and Data Extraction

Identification of publications was conducted by one reviewer (EL). Titles and abstracts were screened by that same reviewer (EL) using the strict inclusion and exclusion criteria. Subsequently, selected articles were assessed in full text and studies corresponding to the eligibility criteria were included in the systematic review.

Data were extracted through a data collection form consisting of the study characteristics, used techniques and the different outcome measures. Study characteristics included author, year of publication, study design, number of patients, patient selection (inclusion criteria and exclusion criteria), procedure information, degree of stenosis, follow-up range, outcome, conclusions, and risk of bias. Interventions included endoscopic procedure with cold knife, dilation (rigid or balloon) or laser (CO_2_ or Nd:YAG), that were used in several combinations and either combined with mitomycin C and/or corticosteroids or used without supplemental therapy. The primary endpoint was the time interval between successive treatments and the secondary endpoints were the rate of recurrence, mean amount of interventions per patient, tracheotomy rate in patients and rate of open surgery.

### Data Analysis

Quality was assessed using Methodological Index for Non-randomized Studies (MINORS) criteria, a validated instrument to detect bias among observational studies, in particular studies concerning surgical interventions. MINORS criteria consist of two sets of criteria, eight items for non-comparative studies and four additional items for comparative studies. The different items were scored with three possible appreciations: 0 (not reported), 1 (reported but inadequate), or 2 (reported and adequate) (9). Based on level of bias, each article was assigned a score. Studies scoring a value above 11 were regarded as having a low level of bias. Studies scoring below 11 were considered to be at risk for a higher level of bias.

The weighted mean was calculated for the different outcome measures in this manner: the product of the reported outcome measure with the number of included patients was determined. The sum of these products was calculated and subsequently divided by the total number of patients included in all series.

## Results

**Figure 1:**
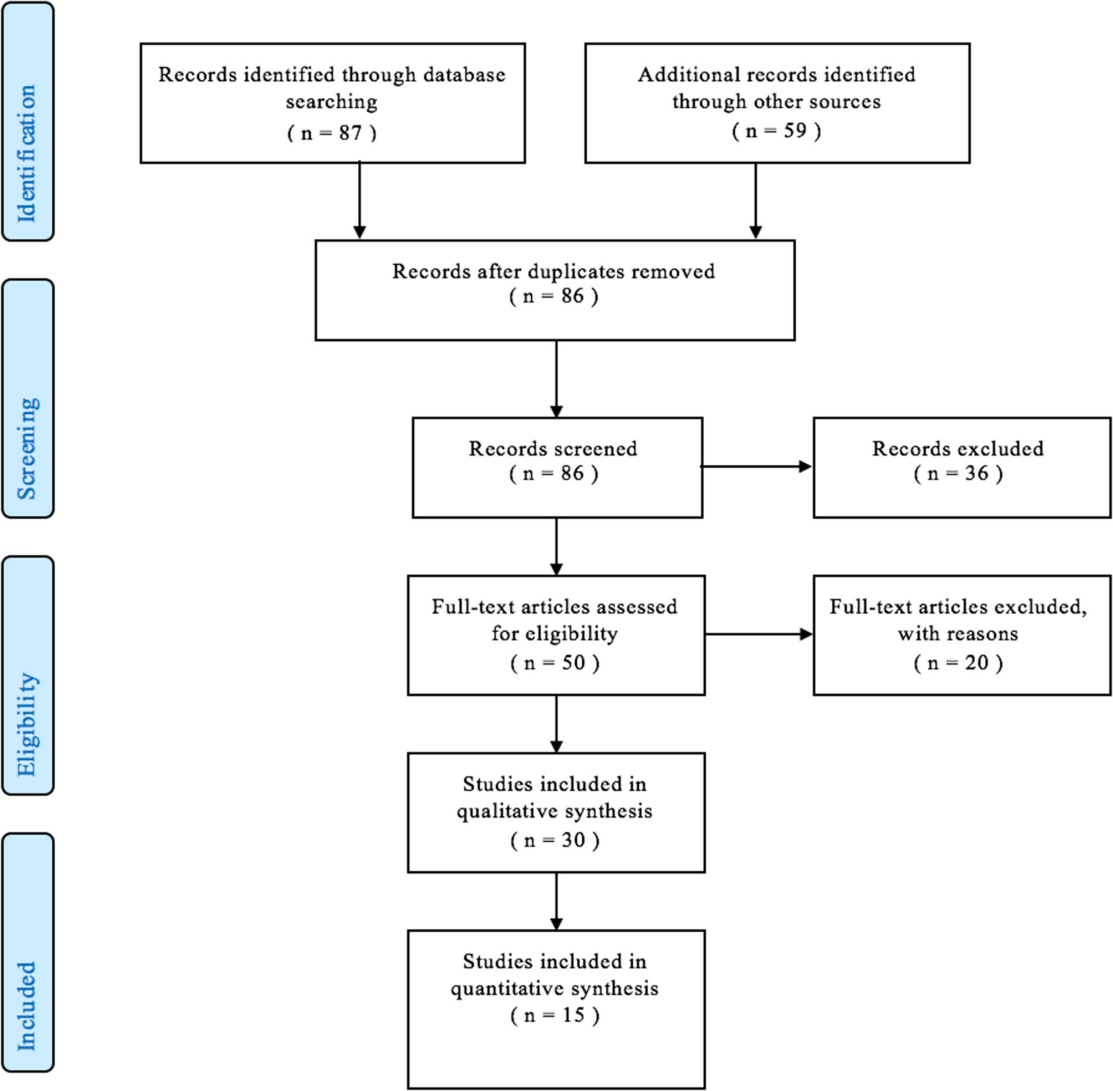
Preferred reporting items for systematic reviews and meta-analyses flowchart showing the resulting articles after implementing inclusion and exclusion criteria.

Eighty-six abstracts were reviewed. Fifty articles were selected for full-text review, and 15 articles were included in the final analysis. The result of the literature search is depicted in a PRISMA flowchart shown in Figure 1.

### Quality Assessment

**Figure 2:**
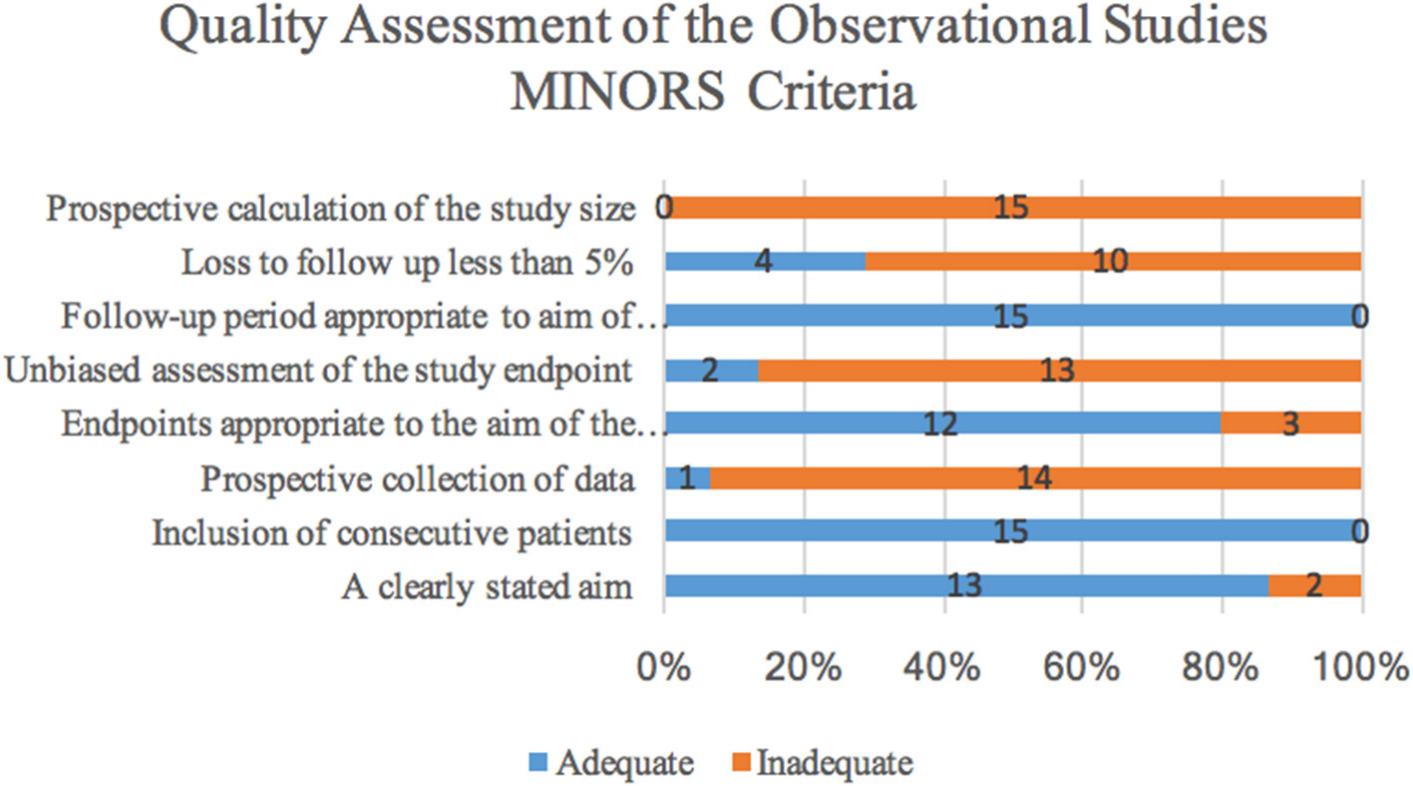
Distribution of the adequacy of MINORS criteria showing the amount of articles scoring adequate or inadequate for the criteria.

**Figure 3:**
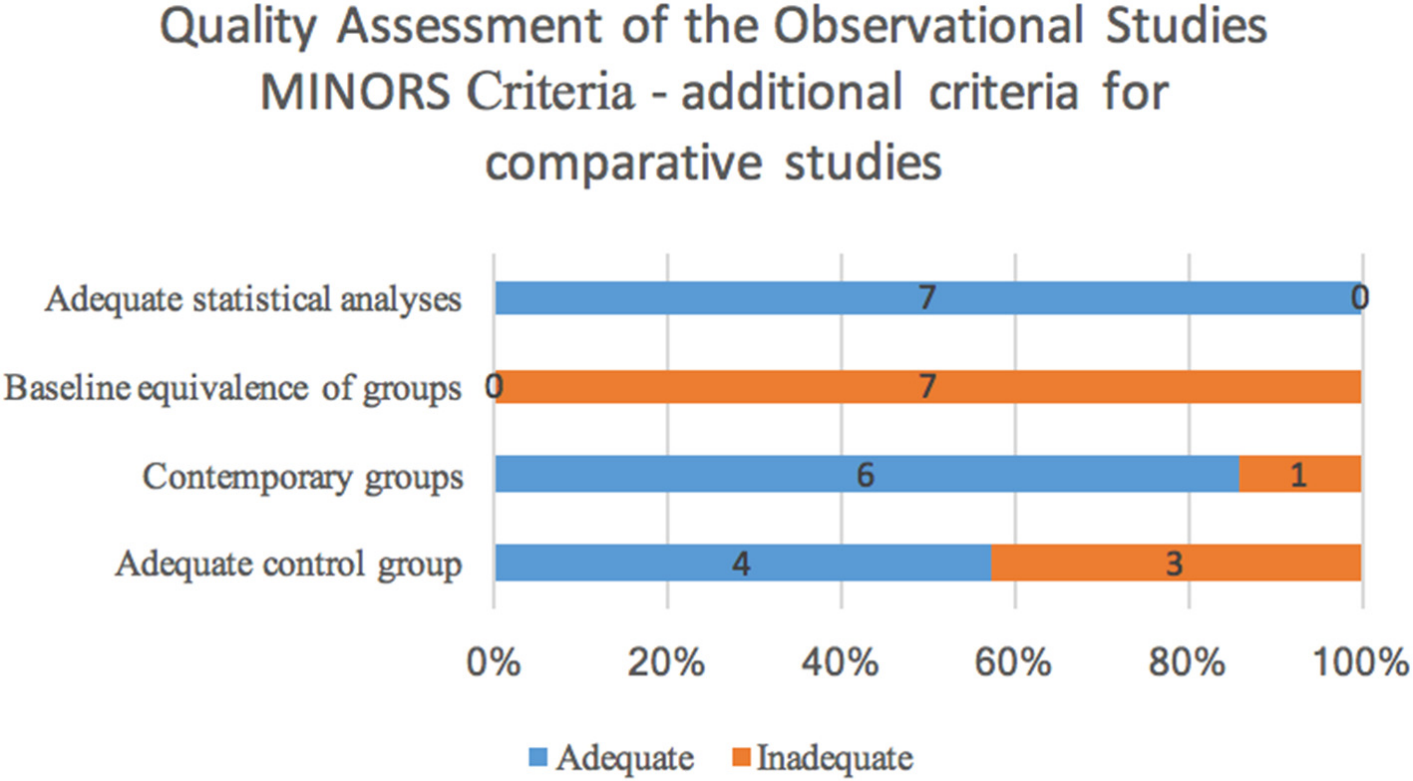
Distribution of the adequacy of the four additional MINORS criteria assessing comparative studies, showing the amount of articles scoring adequate or inadequate for the criteria.

Quality assessment was conducted using the MINORS criteria and a histogram of the distribution of the adequacy of each criterion can be found in Figures 2, 3. Of the 15 studies, 14 were retrospective in design and one was prospective. There were seven comparative studies, and eight non-comparative studies. The methodological items that were most reported were the clearly stated aim, inclusion of consecutive patients, endpoints appropriate to the aim of the study and length of follow-up. However, prospective collection of data, unbiased assessment of the study endpoints, rate of patients lost to follow-up, and prospective calculation of study size were frequently not adequately included. All 15 studies scored below the threshold value of 11 because of the predominantly retrospective design and the low amount of studies mentioning loss to follow-up. We could therefore presume that the selected studies are at risk for bias.

### Patient and Treatment Characteristics

Of the 15 articles, 7 (1, 2, 5, 10–13) (46.7%) discussed the degree of stenosis based on the Cotton-Myer scoring and 5 (5, 13–16) (33.3%) described the severity according to the length of the stenosis. The percentage of stenosis and the location below the vocal folds were discussed in 2 (16, 17) (13.3%) and 2 studies (15, 16) (13.3%), respectively. Another study provided the anterior-posterior, lateral, and cross-sectional dimensions of the stenosis (18). Classification by the Freitag classification was used in 1 study (6.7%) (5). All the articles together included a total of 862 iSGS cases (range 10–384, σ 90.84), of which 846 women and 16 men with a mean population size of 57 subjects. This confirms a clear female preponderance (range 80–100%) with a mean percentage of female patients in the total iSGS population of 96.9%.

The mean age was 48 years (range 36–54 years, σ 4.45). The mean reported follow-up time was 51.2 months (range 16.3–144 months, σ 29.68). The seven studies using the Cotton-Myer scoring reported a grade I stenosis in 11.3% of patients (18/160 patients), grade II in 43.8% (70/160) of patients, grade III in 43.1% (69/160) of patients and grade IV in 2% of patients (3/160) (1, 2, 5, 10–13). The mean length of the stenosis across the studies was 1.3172 cm (range 0.82–1.706 cm, σ 0.35) (5, 13–16).

The pooled outcomes of the surgical techniques were as follows: 444 procedures included cold knife incision and 736 procedures included laser incision, of which 688 CO_2_ laser incision and 48 Nd:YAG laser incision. These incision procedures consisted of radial (generally two to four incisions at 12, 3, 6, and 9 o'clock, where 12 o'clock is anterior) incisions or incisions creating 2 flaps that rotate to partially cover raw surface (18). Five hundred fourteen procedures comprised rigid dilation, 616 comprised balloon dilation and stents were used in merely 14 interventions. In addition, during 251 procedures mitomycin C was administered and during 340 procedures corticosteroids were injected.

**Figure 4:**
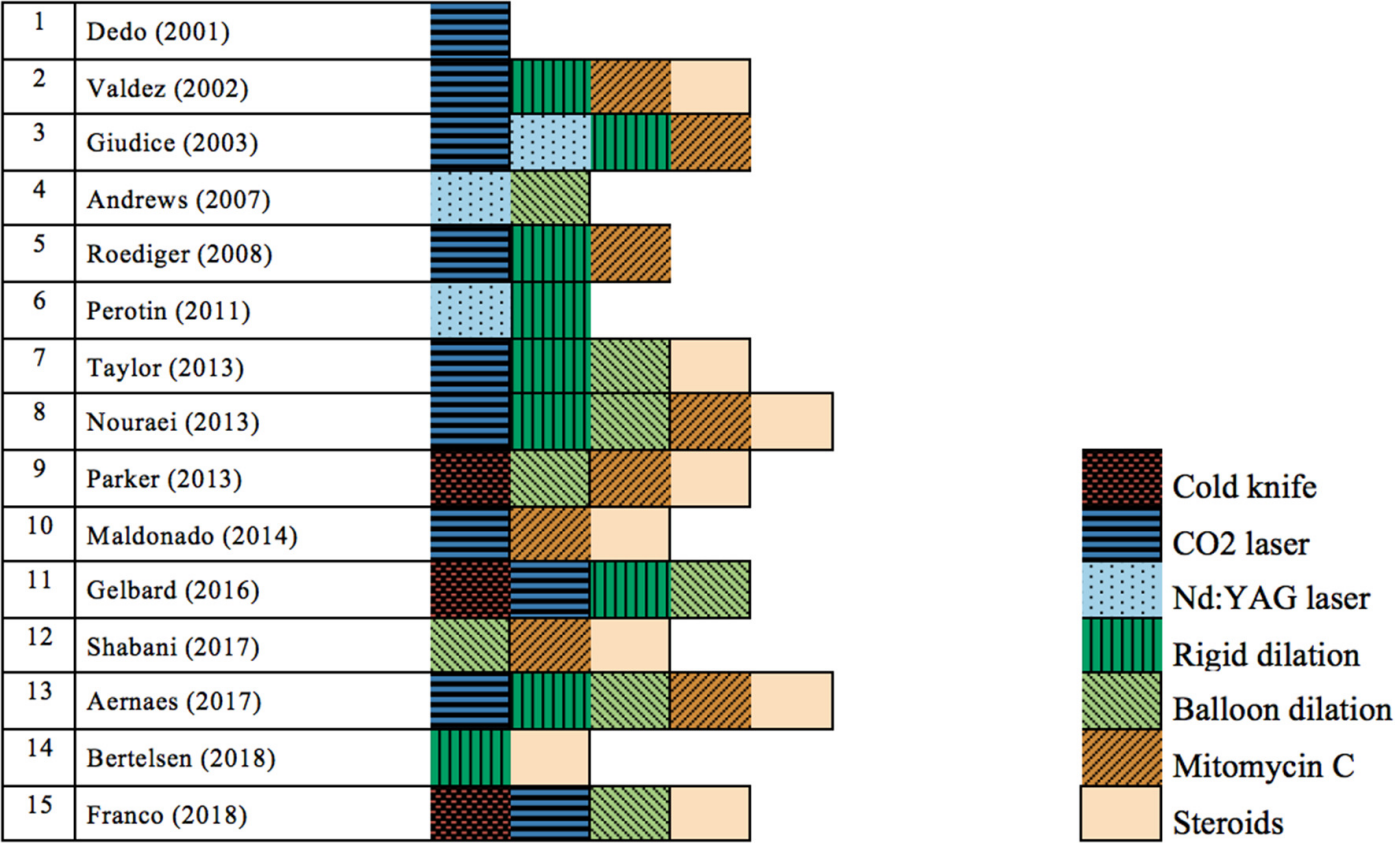
Distribution of the used techniques for treatment of patients with idiopathic subglottic stenosis in the different selected trials, coded by color. CO_2_ laser, carbon dioxide laser; Nd:YAG laser, neodymium-doped yttrium aluminum garnet laser.

When looking into the different techniques used in the 15 selected articles, we determined that every study examined a different set of treatment techniques, except for Nouraei et al. and Aarnaes et al., who both combined CO_2_ laser, rigid dilation and balloon dilation with mitomycin C and corticosteroids (2, 19). However, their study intention was different, since Nouraei et al. conducted a non-comparative study and Aarnaes et al. conducted a comparative study (2, 19). Figure 4 shows a visual overview of the different techniques in the trials. It is striking to note that only 1 trial (6.7%) examined the use of an isolated technique (18) and 14 studies (93.3%) described the use of a combination of techniques (1, 2, 5, 10–17, 19–21). Of the 15 studies evaluated, 3 studies (15, 16, 21) (20%) reported on cold knife technique, 10 (1, 2, 10, 12, 14, 16, 18–21) (66.7%) used CO_2_ laser incisions, 3 (5, 10, 11) (20%) used the Nd:YAG laser, 9 (1, 2, 5, 10, 12–14, 16, 19) (60%) performed rigid dilations and 8 (2, 11, 12, 15–17, 19, 21) (53.3%) balloon dilations. Mitomycin C and corticosteroids as an adjunctive therapy or as a solitary therapy were used in 8 (1, 2, 10, 14, 15, 17, 19, 20) (53.3%) and 9 studies (1, 2, 12, 13, 15, 17, 19–21) (60%), respectively. Several studies reported a disunity in treatment over the course of the study or an intra-population variation in treatment. Heterogeneity in treatment was suspected in nine studies (60%) (1, 2, 5, 10, 12, 14, 16, 17, 19). All other studies do not specifically report a variance in endoscopic treatment (11, 13, 15, 18, 20, 21). Additionally, some patients were treated with airway stenting after the endoscopic procedure, which biases outcome parameters (2, 5).

Considering the vast amount of combination possibilities with the four endoscopic techniques and the two adjunctive treatments, it was judged that there were insufficient similarities such that a quantitative meta-analysis would not produce meaningful results.

### Outcome Measures

Table 1 gives an overwiew of the studies included in this review. Table 2 depicts the range and mean of the different outcome measures.

**Table 1:** Serial number, characteristics, and outcome of the selected articles.

**Study no**.	**References**	**Study design**	**Number of idiopathic patients**	**Procedure information:** **-Cold knife** **- Laser** **- Rigid dilation** **- Balloon dilation** **- Mitomycin C** **- Corticosteroïds**	**Degree of stenosis**	**Duration of follow-up**	**Outcome:** **- Rate of recurrence ** **- Amount of interventions** **- Time interval between interventions** **- Tracheotomy rate** **- Rate of open surgery** **- Amount of complications**	***p*- values**
1	Dedo and Catten (18)	Retrospective chart review	50	Laser: CO_2_ laser	No Cotton- Myer grading measurements: - Average anterior- posterior: 4 mm - Average lateral: 5 mm - Average cross- sectional area: 21 mm^2^	1970–2000 (mean: 71 months)	- Rate of recurrence: 100% - Amount of interventions: 414 (8.28 per patient) - Time interval between interventions: 2 months - Tracheotomy rate: 13 - Rate of open surgery: 3 - Amount of complications: 1	/
2	Valdez and Shapshay (1)	Retrospective chart review	16	Laser: CO_2_ laser Rigid dilation: ventilating rigid bronchoscopes of progressively larger diameters Mitomycin C: 6 patients Corticosteroïds: 8 procedures	Cotton- Myer grading: - Grade I: 4 - Grade II: 10 - Grade III: 2 - 8 patients with short (<1 cm) stenotic lesion - 8 patients with long (> 1 cm) stenotic lesion	Mean: 75.5 months	- Rate of recurrence: 57% - Amount of interventions: 45 (2.95 per patient) - Time interval between interventions: / - Tracheotomy rate: / - Rate of open surgery: 3 - Amount of complications: /	/
3	Giudice et al. (10)	Retrospective chart review	30	Laser: CO_2_ laser: 4; Nd:YAG laser: 26 Rigid dilation Mitomycin C: 6 patients	Cotton- Myer grading: - Grade II: 26 - Grade III: 4	1986–2002 (mean: 55 months)	- Rate of recurrence: / - Amount of interventions: /(2.4 per patient) - Time interval between interventions: / Tracheotomy rate: 6 - Rate of open surgery: 5 - Amount of complications: /	/
4	Andrews et al. (11)	Retrospective chart review	10	Laser: Nd:YAG laser Balloon dilation: Cordis balloon dilation catheters	Cotton- Myer grading: - Grade I: 10	2000–2005 (mean: 22 months)	- Rate of recurrence: 50% - Amount of interventions: 20 (2 per patient) - Time interval between interventions: 8.6 months - Tracheotomy rate: / - Rate of open surgery: / - Amount of complications: 0	/
5	Roediger et al. (14)	Retrospective chart review	10	Laser: CO_2_ laser Rigid dilation Mitomycin C	No Cotton- Myer grading Mean LOS: 1.7 ± 0.6 cm	2004–2008 (mean: 16.3 months)	- Rate of recurrence: 60% - Amount of interventions: 18 (1.80 per patient) - Time interval between interventions: 9 months - Tracheotomy rate: 0 - Rate of open surgery: 0 - Amount of complications: 2	LOS for patients with eccentric stenosis longer than concentric stenosis (*P* < 0.3)
6	Perotin et al. (5)	Retrospective multicenter study with standard form analysis in 9 institutions	23	Laser: Nd:YAG laser, argon laser, or electrocoagulation Rigid dilation	Cotton- Myer grading: - Grade II: 23 Freitag classification: - Grade II: 8 - Grade III: 7 - Grade IV: 8 Mean LOS: 1.3 cm	Mean: 41 ± 34 months	- Rate of recurrence: 57% - Amount of interventions: 42 (1.82 per patient) - Time interval between interventions: 14 months - Tracheotomy rate: 0 - Rate of open surgery: 2 - Amount of complications: 1	No correlation between risk, delay, or number of recurrence and type (*p* = 0.37), severity of obstruction (*p* = 0.11), height of stenosis (*p* = 0.2), distance from the vocal cords (*p* = 0.83), or endoscopic treatment performed
7	Taylor et al. (12)	Retrospective chart review	24	Laser: CO_2_ laser Rigid dilation: Jackson laryngeal dilators, or rigid bronchoscopic dilation Balloon dilation: continuous radial expansion balloons Corticosteroïds	Cotton- Myer grading: - Grade I: 2 - Grade II: 14 - Grade III: 34	2005–2010 (mean: 2.8 years)	- Rate of recurrence: 54% - Amount of interventions: 55 (2.54 per patient) - Time interval between interventions: 16.5 months - Tracheotomy rate: 0 - Rate of open surgery: 6 - Amount of complications: 0	Patients with GPA underwent a mean of 3.53 surgical dilations per patient compared with 2.54 in those with iSGS (*P* = 0.44)
8	Nouraei and Sandhu (2)	Prospective observational study	54	Laser: CO_2_ laser Rigid dilation: stenting Balloon dilation Mitomycin C: 21 patients Corticosteroïds	Cotton- Myer grading: - Grade I: 5 - Grade II: 19 - Grade III: 27 - Grade IV: 3	2004–2012 (mean: 45 months)	- Rate of recurrence: / - Amount of interventions: 249 (3 per patient) - Time interval between interventions: / - Tracheotomy rate: 10 - Rate of open surgery: 15 - Amount of complications: 9	Success rate after five years of follow- up was 18.7% for concomitant glottic and subglottic disease and 87.5% for subglottic- only disease, and (*P* < 0.05; log- rank statistics)
9	Parker et al. (15)	Retrospective chart review	53	Cold knife: Beaver blade Balloon dilation: angiocatheter balloons Mitomycin C Corticosteroïds: triamcinolone	No Cotton- Myer grading Mean LOS: 0.82 cm	2000–2010Mean: 2.9 years	- Rate of recurrence: / - Amount of interventions: 171.6 (3.24 per patient) - Time interval between interventions: 14.59 months - Tracheotomy rate: 1 - Rate of open surgery: 3 - Amount of complications: /	Association between distance from the vocal folds and time interval (*P* = 0.021), farther from the vocal folds required procedures at more frequent intervals
10	Maldonado et al. (20)	Retrospective chart review	110	Laser: CO_2_ laser Mitomycin C Corticosteroïds: triamcinolone topically and fluticasone inhalation therapy	No Cotton- Myer grading	1987–2012Mean: 3.66 years	- Rate of recurrence: 56.9% - Amount of interventions: / - Time interval between interventions: / - Tracheotomy rate: / - Rate of open surgery: 10 - Amount of complications: 0	Trend suggesting a correlation between aggressive medical treatment and decreased rate of recurrence/person/year (relative risk = 0.52, *P* = 0.051)
11	Gelbard et al. (16)	Retrospective chart review	384	Cold knife Laser: CO_2_ laser Rigid dilation Balloon dilation	No Cotton- Myer grading Percentage of stenosis: 62.74% Stenosis length: 1.706 cm	2000–2014Mean: 56.4 months (for endoscopic group: 54.2 months)	- Rate of recurrence: / - Amount of interventions: / (3.7 per patient) - Time interval between interventions: 12.6 months - Tracheotomy rate: 14 - Rate of open surgery: / - Amount of complications: /	Endoscopic surgeries had a significantly higher rate of disease recurrence than open procedures (chi^2^ = 4.09, *P* = 0.043). Secondary analysis showed no relationship between outcome and center endoscopic surgical volume (Spearman *r* = 0.16, *P* = 0.64)
12	Shabani et al. (17)	Retrospective chart review	37	Balloon dilation Mitomycin C Corticosteroïds: triamcinolone or dexamethasone in 13 patients	No Cotton- Myer grading Degree of stenosis pre- dilation: 52 ± 11% Degree of stenosis post- dilation: 14 ± 5%	2003–2015 (12 years)	- Rate of recurrence: 84% - Amount of interventions: 144 (3.8 per patient) - Time interval between interventions: 21.17 months - Tracheotomy rate: 4 - Rate of open surgery: 4 - Amount of complications: /	Interdilation interval was 556 ± 397 days for patients receiving concomitant steroid injection and 283 ± 36 for those who did not (*P* = 0.079).Degree of stenosis at first dilation was inversely related to interdilation interval but again statistically insignificant (*r* = −0.149, *P* = 0.488).
13	Aarnaes et al. (19)	Retrospective chart review	38	Laser: CO_2_ laser Rigid dilation: bougie dilation Balloon dilation Mitomycin C Corticosteroïds	No Cotton- Myer grading	2003–2013 (mean: 5.3 years)	- Rate of recurrence: 79% - Amount of interventions: 132 (3.47 per patient) - Time interval between interventions: / - Tracheotomy rate: / - Rate of open surgery: 0 - Amount of complications: 0	Age at diagnosis had a significant influence on the interval between reoperations (*p* = 0.034), but in general, the interval between operations did not increase over time
14	Bertelsen et al. (13)	Retrospective chart review	10	Rigid dilation Corticosteroïds triamcinolone	Cotton- Myer grading: - Grade I: 7 - Grade II: 1 - Grade III: 2 - Grade IV: 0 Mean LOS: 1.06 cm	2013–2016 (mean: 32.3 months)	- Rate of recurrence: 40% - Amount of interventions: /(4 per patient)- Time interval between interventions: 15.7 months - Tracheotomy rate: 0 - Rate of open surgery: 0 - Amount of complications: 0	/
15	Franco et al. (21)	Retrospective cohort study	13	Cold knife: forceps, scissors Laser: CO_2_ laser Balloon dilation: Boston Scientific controlled radial expansion balloon Corticosteroïds: triamcinolone acetate (Kenalog- 40) 40 mg/mL, dexamethasone (Decadron) 4 mg/mL, methylprednisolone (Solu- Medrol) 40 mg/ mL, and betamethasone (Celestone) 6 mg/mL (descending frequency)	No Cotton- Myer grading	2011–2017Mean: 3 yearsGroup 1: 3.3 yearsGroup 2: 2.7 years	- Rate of recurrence: / - Amount of interventions:/(2.1 per patient) - Time interval between interventions: / Tracheotomy rate: / - Rate of open surgery: 0 - Amount of complications: 0	Statistically significant improvement was seen in %PEF for both groups (SILSI *P* = 0.007, OR *P* = 0.002)

**Table 2:** Range and mean of the different outcome measures.

	**Range (min–max)**	**Mean**
Study population (number of patients)	10–384	57.47
Follow-up (months)	16.3–144	51.19
Rate of recurrence (percentage)	40–100	68.20^†^
Mean amount of interventions per patient	1.8–8.28	3.67^†^
Time interval between interventions (months)	2–21.17	12.62^†^
Tracheotomy rate (percentage)	0–26	7.11^†^
Rate of open surgery (percentage)	0–27.78	10.89^†^
Complication rate (percentage)	0–11	2.48^†^

Figures indicated with

† *represent the weighted mean*.

#### Rate of Recurrence

The reported rate of stenosis recurrence varied between 40 and 100%, with a median of 57%. The weighted mean equaled 68.2%. The highest recurrence rate (100%) was found in a study by Dedo et al., investigating the single use of CO_2_ laser as a treatment modality (18). The combination of techniques with the lowest recurrence rate (40%) was rigid dilation with corticosteroids, described by Bertelsen et al. (13).

#### Mean Amount of Interventions Per Patient

The mean amount of interventions per patient varied between 1.8 and 8.3. The median amount of interventions per patient was 3.0 and the weighted mean equaled 3.4. Again, the highest amount (8.3), with a duration of follow-up of 71 months, was found in the study by Dedo et al. (18). The lowest mean amount of procedures per patient (1.8) was found in the study of Roediger et al., which investigated the combination of CO_2_ laser, rigid dilation and mitomycin C for a follow-up time of 16 months (14).

#### Time Interval Between Interventions

The time interval between interventions ranged from 2 to 21.2 months with a median value of 14.3 months. The weighted mean equaled 12.6 months. The longest time interval (21.2 months) was reported by Shabani et al., using balloon dilation, mitomycin C and corticosteroids (17). The shortest time interval (2 months) was found in the trial by Dedo et al. (18).

#### Tracheotomy Rate

Ten studies reported the tracheotomy rate during follow-up. The tracheotomy percentage ranged from 1.9% (1 out of 53 patients) to 26.0% (13/50), and the weighted mean tracheotomy rate was 7.1%. The highest value (26.0%) was found with the sole use of CO_2_ laser, described by Dedo et al. (18).

#### Rate of Open Surgery

Thirteen studies reported on the rate of open surgery during follow-up because of unsatisfying evolution with endoscopic treatment (1, 2, 5, 10, 12–15, 17–21). In four studies no open surgery during follow-up proved necessary (13, 14, 19, 21). In nine series open surgery was performed, ranging from 5.6% (3 out of 53 patients) to 27.8% of patients (15/54) (1, 2, 5, 10, 12, 15, 17, 18, 20). Therefore, the open surgery percentage varied between 0 and 27.8% and the weighted mean percentage was 10.9%. The highest percentage (27.8%) was reported by Nouraei et al., investigating the combination of CO_2_ laser, rigid dilation, balloon dilation, mitomycin C and corticosteroids (2).

#### Amount of Complications

The amount of complications was described in 10 studies, of which four studies found an amount of complications between 1 and 9 complications in total (2, 5, 11–14, 18–21). Six studies reported that their techniques generated no complications (11–13, 19–21). Nouraei et al. described the highest absolute amount of complications (9 in a population of 54 patients and 249 interventions) in using the combination of CO_2_ laser, rigid dilation, balloon dilation, mitomycin C and corticosteroids (2). They encountered four tracheal infections, two mucosal tears, one distal stent migration, and two incidents of airway crusting. Additionally, one mortality was reported due to “airway infection” (2). Roediger et al. reported two complications in a population of 15 patients receiving 18 interventions. One patient experienced increased pulmonary secretions and one patient experienced severe airway narrowing (14). Dedo et al. reported one postoperative flap swelling requiring tracheotomy for 414 interventions in 50 patients and Perotin et al. described one stent migration requiring replacement for 42 interventions in 23 patients (5, 18). The occurrence of a complication per executed intervention was the highest in the trial by Roediger et al., with a rate of 11%, and lowest in the series by Dedo et al., with a rate of 0.2% (14, 18).

## Discussion

The ideal management of iSGS has not yet been elucidated and remains challenging since well-accepted guidelines are not readily available. In this review, we aimed at identification of the different endoscopic techniques for treatment of iSGS and at evaluation of the obtained treatment results. However, several limitations were identified, making comparison of the different techniques extremely difficult.

The majority of the studies were retrospective in design, which is susceptible to biases associated with non-random assignment of intervention. As the evaluation of data quality using the MINORS criteria suggest, all studies scored below the threshold value of 11, putting the selected studies at risk for a significant bias.

Moreover, the characterization of the stenosis differed between the different trials, some utilizing length of stenosis, others using the Cotton-Myer grading or the Freitag classification. Within a certain study population, different patients might present with a different grade of stenosis, making comparison of the patients between the selected trials difficult a priori. Another obvious confounder of the study results is the possible previous treatment the patient has received before being referred to the hospital conducting the trial. Due to the retrospective design of most selected trials, this feature of the study population is frequently not registered or reported. Moreover, several studies reported a disunity in treatment over the course of the study or an intra-population variation in treatment, changing the combination of techniques or using different doses of adjuvant therapy interchangeably. The majority of the selected studies reported a heterogeneous approach of the patient population. A performance bias, caused by this variance, was suspected in nine trials (1, 2, 5, 10, 12, 14, 16, 17, 19). Six other trials did not specifically report a disunity. However, whenever treatment of recurrence was not specified, it is not possible to rule out potential dissimilar treatment methods for the same patient over the course of the disease. Additionally, motivations for variations in practice among surgeons could not be assessed and it is possible that these differences have resulted in selection bias. Furthermore, there might be unmeasured confounding factors related to intraoperative and postoperative management (6). The comparison of the results was likewise hindered by the variabilities in the outcome measures. The definition of recurrence appeared variable and the majority of the selected trials did not specify the parameters for assessing the need for re-intervention or used subjective criteria, such as recurrence of dyspnea (13, 21). Taking all these limitations into account, following findings need to be interpreted with caution. Only one trial examining a single endoscopic technique was identified, making one by one comparisons of the different techniques impossible. The only selected study evaluating the use of a single technique, CO_2_ laser monotherapy, reported the most unfavorable outcomes: highest rate of recurrence (100%), the highest mean amount of interventions per patient (8.3) and the shortest time interval between interventions (2 months) (18). Moreover, a tracheotomy rate of 26% was reported. These findings suggest that monotherapy with CO_2_ laser incision might be suboptimal for the endoscopic management of patients with iSGS. The combination technique with the highest recurrence rate (84%) proved balloon dilation with mitomycin C and corticosteroid application, described by Shabani et al., while rigid dilation with corticosteroids, described by Bertselsen et al., resulted in the lowest recurrence rate (40%) (13, 17). These results suggest that rigid dilation with corticosteroids might have a better outcome than balloon dilation with corticosteroids. However, this first combination resulted in a longer time interval between procedures when compared to the second combination (21.2 vs. 15.7 months). This suggests that, although the recurrence rate after balloon dilation is high, patients who recur will remain symptom free during a longer time interval compared to the (lower portion) of patients who recur after rigid dilation.

The combination of techniques with the lowest mean amount of interventions per patient (1.8) is the combination of CO_2_ laser, rigid dilation and mitomycin C, described by Roediger et al. (14). This suggests that this combination results in the lowest recurrence of severe dyspnea that necessitates repeat surgical treatment. However, when looking into the time interval between interventions, which was the primary outcome measure of this systematic review, the combination of CO_2_ laser, rigid dilation and mitomycin C was the most unfavorable, 9 months. This discrepancy between a low mean amount of interventions per patient and a short time interval between interventions might be explained by the short duration of follow-up, 16.3 months, of that particular trial. It is important to note that the rate of recurrence is strongly dependent on the duration of follow-up, since a longer follow-up time renders more time to develop restenosis and recurrence of symptoms requiring re-intervention. Mean amount of interventions per patient is heavily biased by duration of follow-up as well and as such, both outcome measures need to be interpreted with great caution. Time interval between interventions is a more objective outcome measure, if re-interventions are not scheduled according to the treating physician's practice. The trial which reported the longest time interval between interventions, 21.2 months, with also the longest duration of follow-up, 144 months, is the trial by Shabani et al., investigating the combination of balloon dilation, mitomycin C and corticosteroids (17).

Tracheotomy rate is also a fairly objective outcome measure for success of endoscopic therapy, since tracheotomy is a salvage therapy that is only performed in an urgent setting caused by respiratory distress. In the selected literature, the tracheotomy rate varied between 0 and 26%, with several trials reporting no need for tracheotomy. The rate of open surgery, however, might be susceptible to a high degree of variability between investigators and surgeons. Since no protocols concerning the treatment of iSGS are available, the switch from endoscopic techniques to open surgery is highly dependent on the self-composed protocols of the hospital and preferences of the treating physicians. This outcome measure is therefore not an independent reflection of the efficacy of a certain endoscopic approach.

Notwithstanding the rapid rise of the endoscopic techniques in the management of idiopathic subglottic stenosis, open surgery such as of laryngotracheal reconstruction or, more important, cricotracheal resection (CTR) still plays an important role in the treatment of iSGS cases refractory to endoscopic treatment. Gelbard et al. investigated the variation between and within open and endoscopic treatment approaches and assessed the disease recurrence in patients with iSGS. Their study population comprised 479 iSGS patients across 10 participating centers and 80.2% were managed endoscopically, whereas 19.8% underwent open reconstruction. The rate of disease recurrence was significantly higher for endoscopic treatment than that for open approach. Only 40% of patients who underwent open surgery recurred by postoperative day 1,000. They also discerned a significant negative correlation between surgical volume and recurrence rate, higher volume was related to less recurrence (16). Axtell et al. reported their experience with single-staged laryngotracheal resection and reconstruction as a definitive treatment of iSGS. They reported a recurrence in 23 (8.7%) patients, as determined by symptoms and bronchoscopic findings (22). Morcillo et al. conducted a retrospective trial including 64 patients who were treated by single-staged laryngotracheal resection, with a success rate of 97% (23).

These data suggest that, with regards to stenosis recurrence, open surgical techniques have superior results compared to endoscopic techniques, but the minimally invasive nature and low rate of complications make endoscopic treatment a good first line treatment for iSGS. However, given the huge variation in combination of endoscopic techniques used as well as all confounding factors and low quality of studies, it is impossible to make hard statements about the “ideal endoscopic technique,” which is an important drawback of this review. After all, every study included in this review is subject to several limitations, such as small sample size, short follow-up, possible previous treatments, inconsistency in combination of techniques used within case series and variation in outcome measures between series. Moreover, the majority of the studies were retrospective in design, making them susceptible to biases associated with non-random assignment of intervention. As a consequence, objective comparison of endoscopic technique combination is impossible and no hard conclusions can be formulated. Ideally, a prospective, randomized controlled trial assessing different endoscopic techniques with a homogenous study population with similar Cotton-Myer grading, similar length of stenosis and similar distance from the vocal fold with an adequate duration of follow-up is merited and would provide more reliable evidence. Outcome measures such as time interval between interventions and tracheotomy rate warrant the most objective clinically applicable results. Parameters for assessing recurrence should be set a priori, both objective (percentage of lumen narrowing on laryngoscopy/bronchoscopy or peak expiratory flow evolution) and subjective (significance of dyspnea and effects on quality of life). When re-intervention is indicated, the treatment combination should be identical to the primary combination throughout the entire study period. Regular reassessment with laryngotracheoscopy/bronchoscopy to detect adverse effects on the highly reactive airway mucosa is also advised. Transition criteria for open surgery should also be set a priori to avoid inter-surgeon variability. The main goal of the proposed trial should be to find the best endoscopic treatment regimen.

## Conclusion

The optimal treatment of iSGS remains unknown and since the rate of recurrence is high, success of treatment is based on reducing disease recurrence and avoiding tracheotomy with a technique causing the least morbidity. This systematic review investigating the outcome of endoscopic management of iSGS included 15 articles. For endoscopic treatment of iSGS, a multitude of different endoscopic techniques in various combinations are currently being used, all with a high rate of recurrence but low morbidity. Since treatment morbidity is highly dependent on the frequency of interventions, time interval between interventions was chosen as the primary outcome measure. In this review, no superior modality could be identified. Endoscopic treatment has shown to be a safe and effective approach for the treatment of iSGS. Consequently, endoscopic management is a valuable primary treatment, but open surgery does still have an important role in the iSGS management, especially in cases with multiple recurrences after endoscopic treatment. The uncovering of the ideal minimally invasive combination of techniques generating an indefinite patent trachea without causing disruptive injury to the tracheal mucosa still requires further investigation.

## Data Availability Statement

The raw data supporting the conclusions of this article will be made available by the authors, without undue reservation, to any qualified researcher.

## Author Contributions

JM was responsible for design of the systematic review, writing, and reviewing of the manuscript. EL was responsible for literature search and writing of the manuscript. PD and GH collaborated in the writing and reviewing of the manuscript.

### Conflict of Interest

The authors declare that the research was conducted in the absence of any commercial or financial relationships that could be construed as a potential conflict of interest.
